# A systematic review of primary school teachers’ wellbeing: room for a holistic approach

**DOI:** 10.3389/fpsyg.2024.1358424

**Published:** 2024-06-10

**Authors:** Mumine Ozturk, Michael Wigelsworth, Garry Squires

**Affiliations:** Manchester Institute of Education, The University of Manchester, Manchester, United Kingdom

**Keywords:** wellbeing, teacher wellbeing, systematic literature review (SLR), primary school teacher, well-being

## Abstract

Although the investigation of mental health and wellbeing in education has shown an exponential increase on an international scale, attention has primarily been paid to students, leaving the concept of teacher wellbeing comparatively overlooked. Extant literature offers numerous divergent descriptions, with some academics even avoiding an explicit definition of the term. Thus, there are limitations and inconsistencies in understanding teacher wellbeing as a unique construct. The aim of the current study was three-fold; (1) to assess the extent to which existing research reflects the multidimensional nature of the term teacher wellbeing, (2) to determine whether a holistic construct of teacher wellbeing could be justified, and (3) to evaluate the methodological quality of studies identified. A systematic review following the PRISMA statement was applied to peer-reviewed papers published between 2016 and 2021. Following the screening of 1,676 studies, this paper reports on findings drawn from a final sample of 61 articles conceptualizing teacher wellbeing. Studies were organized by their dominant discourses, namely negativity/ deficiency, positivity/ flourishing, and/or professionalism. Findings illustrate that teacher wellbeing was primarily conceptualized with a professionalism approach (with 18 of the identified studies taking solely this perspective). This is not completely consistent with the prior work that focused on stress and burnout (negativity/ deficiency approach) while exploring teachers’ mental health and wellbeing. More importantly, there were only 6 studies that considered all three discourses together. This paper argues that important information is lost through neglecting alternative lenses, requiring further attention in order to address teacher wellbeing comprehensively. Such an endeavor is essential for shaping interventions and strategies aimed not only at enhancing teacher wellbeing but also at improving student outcomes and, ultimately, the overall quality of education.

**Systematic review registration:** https://www.crd.york.ac.uk/prospero/display_record.php?ID=CRD42021278549PROSPERO, CRD42021278549.

## 1 Introduction

Although teaching is often described as a rewarding profession, it presents a number of challenges that have been seen to impact teachers’ mental health and wellbeing. Indeed, current findings reveal a troubling scenario with the United Kingdom’s Department for Education (DfE) (2018) suggesting that poor “general wellbeing” is a “main contributing factor in [teachers’] decision to leave the profession” (DfE and CooperGibson Research, 2018, p. 21). Increased reports of teacher mental health concerns, as well as a teacher recruiting and retention problem–and its associated implications such as sick leave and turnover—have further alarmed policymakers and researchers (Brady and Wilson, 2021).

The COVID-19 pandemic has only exacerbated existing concerns. Pandemic pressures have had a particular impact on education (Jones and Kessler, 2020), including increasing job expectations because of uncertainty, workload, negative perception of the profession, concern for others’ wellbeing, health struggles, and multiple roles (Kim et al., 2022). In the UK with the pandemic, 52% of all teachers (50% of all education professionals) felt their mental health and wellbeing had declined either considerably or a little (Savill-Smith and Scanlan, 2019, p. 18).

Despite alarming indications of poor mental health for teachers, alternative perspectives on teachers’ wellbeing exist, such as the positive psychology approach, which emphasizes individuals’ control over their own wellbeing. However, there has been criticism of positive psychology’s emphasis on the individual, given limited evidence regarding the extent to which individuals can influence their wellbeing compared to the influence of their life circumstances (Brown and Rohrer, 2019). The primary discourses in the field, including positive psychology and negative health-deficient approaches, will be further examined in the following sections. However, it is vital to acknowledge that overlooking alternative perspectives here may hinder our ability to fully tackle issues like teacher retention in the profession. Despite limitations and inconsistencies in understanding teacher wellbeing, as cited in Cann et al. (2023), scholars have urged the field to expand beyond individual-focused approaches, recognize the impact of context on wellbeing, and embrace greater complexity (Kern et al., 2020; Lomas et al., 2020). Ultimately, a compelling necessity emerges to gain a clear and comprehensive understanding of the term (Ozturk, 2023).

To deepen our understanding of the concept of teacher wellbeing, the present study delves into a comprehensive conceptualisation of primary school teachers’ wellbeing. Primary teachers face unique stressors and strengths compared to their counterparts. Clear data indicates that teacher stress was intensified among primary school teachers and special needs teachers who offer more support and input to students than other teachers (Agyapong et al., 2022). The increased stress among primary school teachers may be attributed to the additional time and energy invested in younger students who may require more support. In this study, our focus is on mainstream primary school teachers. In Warnes et al.’s (2022) study, the term mainstream schooling was defined as, “a system to provide all students, regardless of any challenges they may have, with access to age-appropriate general education in their locality to enable them to reach their potential” (p. 34).

A clear understanding of the concept of teacher wellbeing is crucial to contextualize existing research. Therefore, the primary aim of this systematic review is to evaluate the extent to which existing research on teacher wellbeing reflects its multidimensional nature. While focusing on mainstream primary school teachers’ wellbeing, we also aimed to assess the methodological quality of the existing research. The methodological quality assessment of primary studies included within systematic reviews can offer an indication of the strength, reliability, and repeatability of the evidence upon which the conclusions of the review are founded (Higgins and Green, 2011). With these aims in mind, the upcoming sections will begin by providing contextual information about the definition and conceptualisation of teacher wellbeing. Subsequently, we will examine previous reviews in the field, and then summarize the details of the current study, including the research questions.

### 1.1 Defining wellbeing

The World Health Organization (WHO) defines mental health as “a state of well-being in which an individual can realize his or her own potential, cope with the normal stresses of life, work productively and make a contribution to the community” (WHO, 2016). However, describing wellbeing is challenging since the meaning of the construct may alter in many aspects, such as intrapersonal or interpersonal construct, construct subjectivity or objectivity, and construct stability through time (being a state or a process) (Ereaut and Whiting, 2008).

As a nature of this multidimensional construct, there are multiple theoretical frameworks and conceptualisations in the literature. Some of the widely acknowledged approaches to defining and conceptualizing wellbeing are as follows: hedonic wellbeing (e.g., happiness and life satisfaction) (e.g., Diener, 1984); eudaemonic wellbeing (e.g., positive psychological functioning and living life to the full) (e.g., Ryff, 1989); human flourishing (e.g., open, engaged, and healthy functioning) (e.g., Ryan and Deci, 2011); subjective and objective wellbeing, etc.

From these ideas, objective measures of wellbeing point to facets of the physical world, such as the existence of financial resources, while subjective indicators report on personal experiences of wellbeing, which are inherently private and challenging to quantify (Manning et al., 2020). Although objective and hedonic wellbeing understandings are useful for measurement, these approaches were criticized for underestimating the complexity of what it means to “be-well” (Brady and Wilson, 2021). Subjective eudaemonic approaches, on the other side, acknowledge the complexities of wellbeing and add to the increased popularity of positive psychology approaches.

Positive psychology, which some scholars (e.g., Dodd et al., 2021) regard as combining the hedonic and eudemonic approaches, prioritizes individuals’ experience and self-knowledge. From the positive psychology perspective, wellbeing is conceptualized as a positive emotion, engagement, relationships, meaning and achievement (referred to as the PERMA model from the acronym of the components) (Seligman, 2011). Positive psychology acknowledges that individuals and their interactions are integrated in a social sense and attempts to understand and learn how to create positive institutions (Seligman and Csikszentmihalyi, 2000).

However, positive psychology is not without critics. For instance, according to Wright and Pascoe (2015), the field’s Westernized (culturally established and determined) and individualistic approach ignores the situated and relational aspects of wellbeing. Together with this, Uchida et al. (2015) point out that people define wellbeing differently depending on their culture. They distinguish, for example, between an East Asian view of wellbeing (derived from social harmonies including responding to social expectations and meeting relational obligations) and a European-American view of wellbeing (derived from individual achievement and self-esteem). All these critics and statements bring us—not surprisingly, wellbeing is a complex and multidimensional term. In the literature, there are different understandings of wellbeing and there is yet no single agreed-upon definition and conceptualisation of wellbeing.

Reflecting on the notion of “teacher wellbeing,” it becomes evident that its scope extends far beyond the confines of a mere professional element. To truly grasp the holistic essence of teacher wellbeing, one must transcend the boundaries that traditionally separate personal and professional realms. Teachers are not solely defined by their roles in the classroom; they are individuals with multifaceted lives that interweave the personal and the professional. Furthermore, Warr (1999) describes the relationship between job-specific and context-free wellbeing as a bidirectional nature -“feelings at work and feelings outside work influence each other in a mutual fashion” (p. 399). Consequently, it is crucial to understand not only the professional factors that contribute to teacher wellbeing, but also the external determinants that can affect their wellbeing.

The various conceptualisations of wellbeing have an influence on the concept of teacher wellbeing. Similarly to wellbeing, teacher wellbeing is also a broad concept with several different definitions (von der Embse and Mankin, 2020). All of these make teacher wellbeing a unique phenomenon, distinct from general models but informed by them. Therefore, it should consider all the complexities that wellbeing already has, in addition to other factors that come from the profession itself.

### 1.2 Teacher wellbeing

While academics have described workplace wellbeing as one of the key variables of an individual’s overall wellbeing (Rath and Harter, 2010), there is still little agreement as to the nature and content of the phenomenon referred to as “teacher wellbeing.” With respect to this value as a unique construct, distinct from otherwise generic models of wellbeing, there are identifiable factors unique to the profession. To illustrate, Aelterman et al. (2007) define teacher wellbeing as:

a positive emotional state, which is the result of harmony between the sum of specific environmental factors on the one hand, and the personal needs and expectations of teachers on the other hand (p. 286).

More recently, Acton and Glasgow (2015) synthesize literature on the topic of teacher wellbeing in the neoliberal context and provide another definition that recognizes its hedonic, eudaemonic and relational aspects:

Teacher wellbeing may be defined as an individual sense of personal professional fulfillment, satisfaction, purposefulness and happiness, constructed in a collaborative process with colleagues and students (p. 102).

These examples reflect the situation in approaches to teacher wellbeing; many parallels come from general models of wellbeing, but there are also significant differences that come from the unique nature of the profession such as emphasis on relationships with students, parents, etc. Although there are differences between these definitions, still, both of these definitions have been utilized by others in their studies, for example, Soini et al. (2010), Yildirim (2015), and Brady and Wilson (2021). However, wider literature offers numerous descriptions, with some academics avoiding an explicit definition of the term (Field, 2019). In those situations, it is arguably difficult to understand how the researchers approach teacher wellbeing—for instance, whether they emphasize solely the professional element, only consider negative aspects, or approach it holistically—acknowledging negative, positive and professional aspects altogether.

In addition, jingle-jangle fallacies need to be addressed. Mainly, the jingle fallacy, which holds that scales with the same name measure the same construct, and its opposite, the jangle fallacy, which holds that scales with various names measure various structures (Marsh, 1994). Although this issue is not the main aim of this paper, it highlights that there is a need in the literature to evaluate more critical interpretations of the conceptualisations of teacher wellbeing. However, despite the challenges in defining teacher wellbeing, recognizing its pivotal role in sustaining the profession and influencing student outcomes has prompted efforts to conceptualize it in a measurable and analysable manner (Cann et al., 2020). With all those in mind, the remainder of this section will examine dominant teacher wellbeing discourses, namely negativity/ deficiency, positivity/ flourishing, and/or professionalism (see [author], under review).

#### 1.2.1 Teacher wellbeing: negativity/ deficiency, positivity/ flourishing, and professionalism

In the past, teacher wellbeing has frequently been defined and investigated from a negative viewpoint (Roffey, 2012). Despite recent increases in the use of positive psychology values in research and education, researchers continue to use the term wellbeing as a synonym for stress, burnout, and mental health (Field, 2019). Even though several studies have been conducted on this topic, the term “teacher wellbeing” continues to be used interchangeably with several other concepts in the literature.

More recently many intra-personal variables, including professional wellbeing, self-efficacy, job satisfaction, and acknowledgment, have been recognized as being associated with teachers’ experiences of burnout and stress feelings (Aelterman et al., 2007), suggesting that there are several overlapping factors (and even maybe some of them are jingle-jangle fallacies) affecting teachers’ wellbeing as well as discrepancies. Although there are overlaps between established approaches, none of them has all the relevant elements of teacher wellbeing (Ozturk et al., under review). Therefore, there is an opportunity to explore the conceptualisation of teacher wellbeing systematically.

Recently, Ozturk et al. (under review) examined different concepts of teacher wellbeing, moving toward a unified model of teacher wellbeing. They argued three main discourses in the field: conceptualisation of teacher wellbeing as negativity/deficiency; conceptualisation of teacher wellbeing as positivity/flourishing; and conceptualisation of teacher wellbeing as professionalism. The remainder of the section will introduce these ideas briefly.

##### 1.2.1.1 Negativity deficiency

Teacher wellbeing has historically been approached from a negative/deficit standpoint, which is unsurprising given the prevalence of burnout, stress, and anxiety in the teaching profession, as frequently highlighted in scholarly literature and reports. A discourse of negativity/ deficiency defines teacher wellbeing concerning feelings of stress, burnout, anxiety, depression, etc. (Ozturk et al., under review). For instance, Jennings and Greenberg (2009) developed the prosocial classroom model which suggests that teachers’ wellbeing is closely related to students’ school performance and happiness. Accordingly, teachers who have trouble managing their emotions may eventually develop the first factor of burnout, emotional exhaustion, which can lead to a “burnout cascade” when prolonged (Jennings and Greenberg, 2009). According to this theory, as teachers become stressed, they may become uncaring, insensitive, and show less empathy toward students as a result of this cascade.

When teacher wellbeing is examined through the lens of negativity or deficiency, it sheds light on the critical challenges teachers often encounter in their professional lives. This perspective draws attention to the stressors, burnout, and emotional exhaustion that can negatively affect teachers as they navigate the demanding landscape of schools and education systems. Recent evidence from Finland (Taajamo and Puhakka, 2020), the UK (Scanlan and Savill-Smith, 2021), and the USA (Herman et al., 2020) indicates that teachers’ work-related stress is high globally (as cited in Zhang et al., 2023). It underscores the urgent need for systemic support, resources, and strategies to address these deficiencies and shortcomings, which can have detrimental effects on both individual teachers and the overall quality of education. Although exploring teacher wellbeing in this context provides an opportunity to confront and rectify the wellbeing crisis in the schools, that is not enough to explore teacher wellbeing holistically.

##### 1.2.1.2 Positivity/flourishing

In contrast to a negativity/ deficiency discourse, some researchers (Kun and Gadanecz, 2019; Turner and Theilking, 2019) conceptualize teacher wellbeing by taking into account the positivity/ flourishing approach which refers to the experience of positive emotions, positive relationships, and self-efficacy (Ozturk et al., under review). From this perspective, for instance, Turner and Theilking (2019) attempt to understand teacher wellbeing and its effects on teaching practice and student learning with the help of the PERMA (Positive emotion, Engagement, Relationships, Meaning and Achievement) framework (Seligman, 2011).

Teacher wellbeing in line with positive psychology emphasizes positive aspects of wellbeing over negative/deficit views. This approach focuses on teachers’ strengths or intrinsic resources linked to wellbeing. It is worth noting that positive psychology has been criticized for ignoring the contextual factors that influence the quality of implementation in the real-life setting of a school (Ciarrochi et al., 2016). This brings us to consider that teacher wellbeing goes beyond single aspects and is viewed as a complex construct.

##### 1.2.1.3 Professionalism

The professionalism approach, which focuses entirely on the domain of work, is one typical method of conceptualizing teacher wellbeing (see Collie et al., 2015; Ozturk et al., under review). In this approach, teacher wellbeing is mainly conceptualized through concepts of self-efficacy, job satisfaction, work engagement, authority, and competence. For example, Aelterman et al. (2007) state self-efficacy, workload, and peer support as the main determinants of professional wellbeing in primary school teachers according to their empirical study. Similarly, Yildirim (2015), identifies the main determinants of teachers’ professional wellbeing as self-efficacy, job satisfaction, and recognition. Yildirim (2015) also stated that the most cited measures are self-efficacy and job satisfaction, and the others are authority, recognition, and aspiration.

As stated above, teacher wellbeing is typically examined through the lens of negativity (see Jennings and Greenberg, 2009; Roffey, 2012). Although most recently positive psychology has gotten more attention, both approaches are handling wellbeing as a spectrum. It seems there is a need for a holistic approach that considers every aspect, including the profession itself.

As the professionalism approach emphasizes the occupational aspect of teacher wellbeing, a unique aspect of this approach is to highlight the connections between teacher wellbeing, teachers’ practice, and the impact on the school system and students (Cann et al., 2020). However, the wellbeing of teachers at school is not solely relevant to the professional context (Aelterman et al., 2007), and therefore, further approaches are required in order to fully capture this concept.

### 1.3 Previous reviews

Current research directions vary due to a lack of consensus on defining teacher wellbeing, which is an inherently complex and multidimensional concept. This complexity complicates efforts to achieve a holistic conceptualisation. The concept of teacher wellbeing encompasses various definitions (von der Embse and Mankin, 2020), pointing to conceptual pluralism in the existing literature. Therefore, the field exhibits overlaps and discrepancies, highlighting the need for systematic reviews. For instance, burnout, engagement, and self-efficacy are some of the most common factors in the conceptualisations of teacher wellbeing (Ozturk et al., under review). More than a decade ago, in their review, Spilt et al. (2011) verified that the term wellbeing has largely been examined through a focus on stress and burnout in the literature on teachers.

There are other reviews in the literature that focus on a specific group of teachers or a specific context in order to give a deeper insight into teacher wellbeing. For example, Cumming (2017) reported on early childhood educators’ wellbeing structured according to four themes: work environment, workplace relationships, job satisfaction, and psychological and emotional wellbeing. At the same time, teacher wellbeing has been studied across all educational age ranges, but data on primary school teachers’ wellbeing is relatively limited (see McCallum et al., 2017). To fully comprehend the issue in relation to the sustainability of the profession, we need to investigate that more. Moreover, to the authors’ knowledge, primary-level teachers’ wellbeing has yet to be systematically researched.

In another systematic review, Acton and Glasgow (2015) examined how teacher wellbeing has been articulated, explained, and investigated in neoliberal contexts marked by standards, accountability, and assessment. These studies examined teachers’ wellbeing with a particular emphasis on flourishing in educational settings. Conducting systematic reviews for specific educational settings (like Acton and Glasgow, 2015) is valuable but examining teacher wellbeing in a comprehensive way is also needed. Despite the critical importance of these reviews, a broad systematic examination of teacher wellbeing is still lacking.

There is a continuing discussion about the domains that determine teacher wellbeing and what should be included in each. In other words, the line between what forms a component of teacher wellbeing and what influences teacher wellbeing is unclear (Cann et al., 2020). For instance, according to Viac and Fraser (2020), cognitive wellbeing refers to the range of skills and abilities teachers need to perform effectively, which includes self-efficacy, while McCallum et al.’s (2017) review identifies self-efficacy as a factor impacting teacher wellbeing. Moreover, McCallum et al. (2017) articulate that there have been differing understandings of the term “wellbeing” in general, and “teacher wellbeing” in particular. These findings strengthen the fundamental rationale and relevance of the current study’s case for conducting a comprehensive review of how teacher wellbeing is conceptualized in the literature. With respect to all of these, in this review, we applied many keywords not solely wellbeing. We believe this helped to conduct a comprehensive review of teacher wellbeing.

More recently, Hascher and Waber (2021), reviewed papers that explicitly addressed teacher wellbeing. They restricted their search keywords to wellbeing solely. Their rationale for doing this is to avoid conflating teacher wellbeing with related concepts such as resilience. Against this perspective, we decided to take a holistic vision since teacher wellbeing is a multidimensional construct. As a result, the necessity of the current examination becomes more apparent because it uses a much more comprehensive approach than the previous reviews.

A notable omission from prior reviews is an examination of the methodological quality of the included studies. Assessing the quality of evidence contained within a systematic review is as important as analyzing the data itself. Poor quality studies are at risk of bias, and although skew is typically reflected in the nature of results (e.g., incorrectly identifying a significant effect) poor methodological quality can also be an indication of poor conceptual quality, influenced by the underlying epistemological stance of the research (Howe, 2009). Given the aforementioned plurality of discourses, there is an opportunity to assess whether there are systemic issues in research quality relating to the multidimensionality (and multidisciplinary) of teacher wellbeing research.

Given methodological quality can be influenced by the underlying epistemological stance and considering previous reviews have exhibited shortcomings such as failing to assess the methodological quality of the included papers, the present review is necessary. Assessing the methodological quality of the included papers would provide insights into areas needing improvement in terms of reporting and the extent to which empirical literature investigates wellbeing from specific perspectives. A more comprehensive approach with quality assessment could be advantageous for gaining a deeper understanding of the literature.

In summary, as far as the authors are aware, there is currently no dedicated systematic review focusing on the wellbeing of primary school teachers nor have, prior reviews in this area undertaken an assessment of the methodological quality of the existing literature. These observations collectively highlight a gap in the exploration of how the conceptualisation of wellbeing within the specific context of primary school teachers.

### 1.4 Current study and research questions

The current study investigates the conceptualisation of teacher wellbeing, specifically the conceptualisation of mainstream primary school teachers’ wellbeing. This systematic review also seeks to determine the quality of studies underpinning research. Thus, with this review, we provide important information in order to address teacher wellbeing at a fundamental and critical level. The study investigated the following research questions:

(1) To what extent does the current research on teacher wellbeing reflect the multidimensional nature of the term “teacher wellbeing?”
(2) Are there grounds to advocate for the adoption of a holistic approach to teacher wellbeing?
(3) What is the methodological quality of current research on teacher wellbeing?

## 2 Materials and methods

A review to explore the meaning or interpretation of a phenomenon is likely to adopt a generally configurative logic (Kerres and Bedenlier, 2020). Configurative reviews are more likely to be interested in identifying patterns provided by heterogeneity instead of homogeneity (Gough et al., 2012). This research, therefore, combined various forms of data as a component of the configurative review. On the other hand, in developing the systematic review process, we utilized existing terminology to categorize forms of teacher wellbeing into major discourses, meaning that resulting in an aggregative approach. As different research methods and designs were also included, this review ultimately adopted a mixed method, mixed research synthesis review (Sandelowski et al., 2012). The steps for conducting a systematic literature review are a multi-stage process, following the main steps, as illustrated in Figure 2, outlined by Gough et al. (2013).

**FIGURE 1:**
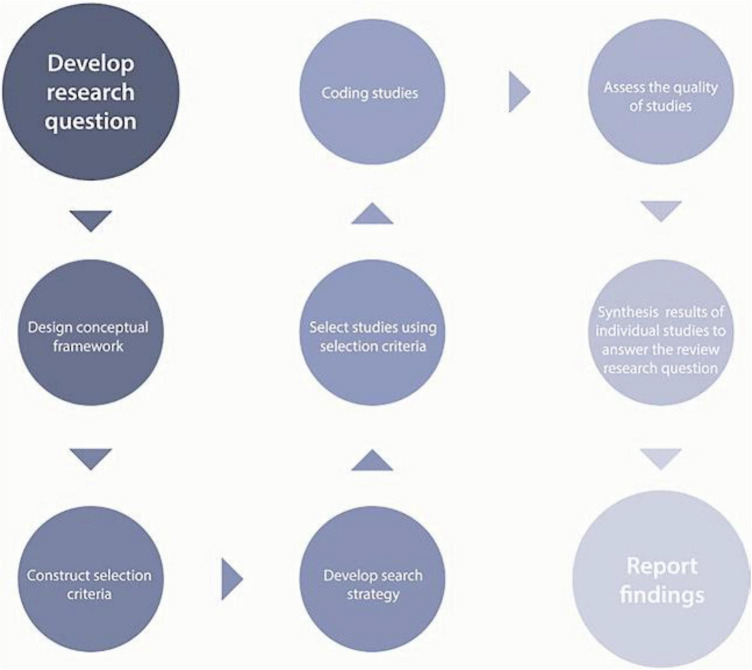
The systematic review process (source: Kerres and Bedenlier, 2020).

**FIGURE 2:**
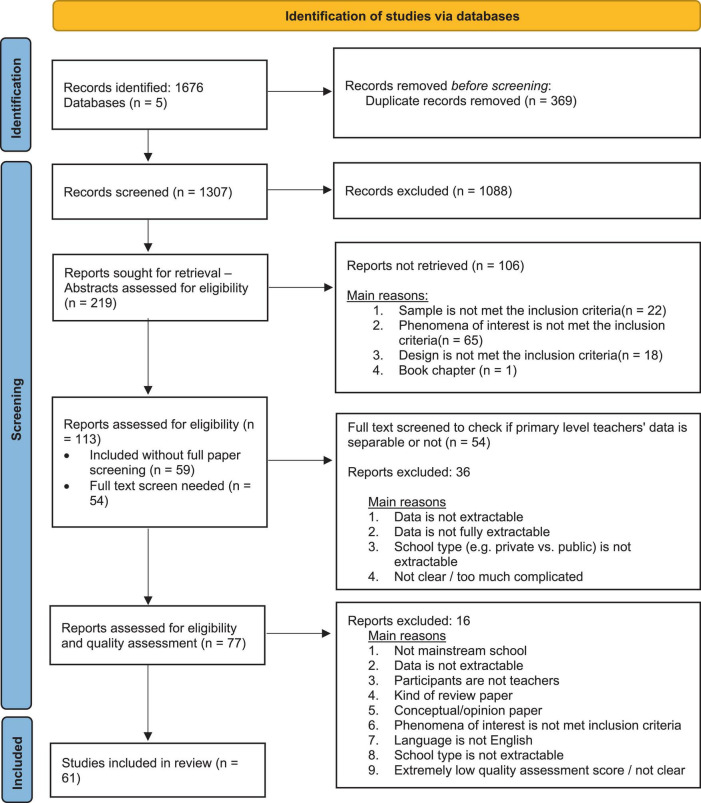
PRISMA flow diagram (source: Page et al., 2020).

### 2.1 Review process

The current study was informed by the Preferred Reporting Items for Systematic Reviews and Meta-analyses (PRISMA) guidelines (Moher et al., 2009). Early and continuing consideration of the PRISMA monitoring checklist elements ensured that the present analysis has been planned, completed, and reported for comprehensive reviews according to accepted best practices. A protocol was published on PROSPERO in October 2021 (registration number: CRD42021278549) and was updated periodically to reflect the progress of the review.

#### 2.1.1 Eligibility criteria

Studies were included in the review if they met the following main inclusion criteria:

• Peer-reviewed journal articles written in English
• Participants were primary school teachers, either exclusively or as part of broader samples if results were presented for subgroups (e.g., primary-level teachers from a sample of mainstream school teachers)

Studies were excluded if eligibility criteria were not met, if the article was not published in English, or if they were not reported in sufficient detail to assess eligibility and compare studies. Other main exclusion criteria:

• Participants from other areas and/or levels, teacher trainees/pre-service teachers and other educational staff members
• Conference papers/proceedings, books, book chapters, reports and other “gray” literature

“Gray” literature was excluded on the basis of a lack of clear guidance for a comprehensive, transparent and reproducible process in including non-peer review sources (Mahood et al., 2014).

In accordance with the inclusion and exclusion criteria, this review focuses on papers that investigate teacher wellbeing in relation to mainstream primary school teachers. But we did not apply only this concept as a search term, we made an effort to be comprehensive, so we included more than 20 terms. The main reason for that this review investigates the conceptualisation of teacher wellbeing comprehensively. The main syntax of the search process:

Elementary OR primary AND teacher* OR educator* AND wellbeing OR well-being OR workload OR burnout OR stress OR recognition OR “job satisfaction” OR self-efficacy OR autonomy OR competence OR support OR “positive emotion” OR engagement OR relationships OR meaning OR accomplishment OR health OR happiness OR interaction OR hope OR resilience OR optimism

Limit to: Peer reviewed, English, Scholarly Journals

Five different databases were searched: PsychInfo; Scopus; Education Resources Information Centre (ERIC); British Education Index (BEI); Applied Social Sciences Indexes and Abstracts (ASSIA). The search is mainly limited to peer-reviewed papers published within the last five years (2016-2021). To examine the contemporary context of the current literature, it was decided to concentrate on the last five years. Results can be seen in Figure 2.

#### 2.1.2 Study selection process

Duplicates were removed and search results were narrowed down through basic filtering. Basic filtering was applied by reading the title (and/or abstract in cases where the title was not sufficient) to discard irrelevant hits (i.e., articles not related to teacher wellbeing in primary schools). The abstracts and keywords in the remaining articles were used to identify any that did not meet the inclusion criteria.

There were three reviewers in total. The first reviewer searched for information sources independently and assessed identified studies for inclusion. Grading of each eligibility criterion was made as “eligible/not eligible/might be eligible (full-text screen needed).” The full text was obtained for abstracts with insufficient information or in a situation of indecision. A study was included when it satisfied the inclusion criteria from the full text. A second reviewer independently cross-checked approximately 30% of papers and if needed, helped to evaluate any lack of clarity in the decision of the first reviewer. To provide consistency between reviewers, an instruction sheet was developed. This sheet was developed as a SPIDER framework (see Supplementary Appendix 1). All disagreements or ambiguities were resolved through discussion with all reviewers.

All tools and processes were piloted before use. The first reviewer extracted the data independently and the second reviewer independently checked the data for consistency and clarity. The third reviewer helped to check reliability. At this stage, all reviewers independently screened 10% of 219 papers, and the results were compared. Inter-rater reliability was 63.6%. In other words, there was over sixty per cent agreement among all three reviewers.

#### 2.1.3 Data collection process

Initial data analysis involved extracting key attributes. Information on authors, abstracts, keywords, and publication dates for each reviewed study was extracted. Data extracted includes the following summary data: study design, sample characteristics, phase of education, quality, and outcomes. All data items were listed, and abstracts were checked to define these variables.

#### 2.1.4 Quality assessment

As per PRISMA guidelines for systematic reviews (Moher et al., 2009), the methodological quality of each article was assessed. In addition to all the stages of the systematic literature review process, the systematic synthesis of research evidence (e.g., appraising study quality and relevance) (Gough, 2007) was considered in the appraisal of evidence. At first, it had been decided that no single study would be excluded because the researcher deems that the quality of a study is low or that there are methodological or other flaws—this would bias the review (Linnenluecke et al., 2020). Yet only one study based on the quality assessment was excluded due to significant difficulties in comprehending the content and purpose of the text.

In total, 77 articles were assessed for quality using a checklist adapted from Croucher et al. (2003) (Table 1) and were deemed to have met essential quality requirements. The adapted checklist contains 10 items: 6 items for essential quality requirements, such as, “Is the research question clear?,” and 4 items for desirable quality requirements, such as “Is the theoretical or ideological perspective of the author (or funder) explicit, and has this influenced the study design, methods or research findings?” The criterion for ethics was assessed as an essential criterion since the main object of the included studies is teachers’ wellbeing which may be a sensitive topic. Each item was scored as Yes (x) or No (left blank). Applying this checklist was important because clear and transparent reporting was required to be able to interpret and critically appraise results and draw comparisons between studies. The results of the quality assessment are presented in Table 1.

**TABLE 1:** Quality assessment.

Included studies	Quality appraisal criteria									
	E	D	E	D	E	E	E	D	D	D*
	Question	Theoretical perspective	Study design	Context	Sampling	Data collection	Data analysis	Reflexivity	Generali-zability	Ethics
	Is the research question clear?	Is the theoretical or ideological perspective of the author (or funder) explicit, and has this influenced the study design, methods or research findings?	Is the study design appropriate to answer the question?	Is the context or setting adequately described?	(Qualitative) Is the sample adequate to explore the range of subjects and settings, and has it been drawn from an appropriate population? (Quantitative) Is the sample size adequate for the analysis used and has it been drawn from an appropriate population?	Was the data collection adequately described and rigorously conducted to ensure confidence in the findings?	Was the data analysis adequately described and rigorously conducted to ensure confidence in the findings?	Are the findings substantiated by the data and has consideration been given to any limitations of the methods or that may have affected the results?	Do any claims to generali-zability follow logically, theoretically and statistically from the data?	Have ethical issues been addressed and confidentiality respected?
Shaukat and Nazir (2017)	x		x	x	x		x		x	
Al-Asadi et al. (2018)	x		x	x	x	x	x	x	x	x
Fareza and Tentama (2020)	x	x	x	x			x		x	
Abdullah and Ismail (2019)	x	x	x	x	x		x		x	
Jalali and Heidari (2016)	x		x	x	x		x		x	
Smetackova et al. (2019)	x	x	x	x	x	x	x	x	x	x
Yunarti et al. (2020)	x		x	x	x		x	x	x	
Daniilidou et al. (2020)	x		x	x		x	x	x	x	x
Edinger and Edinger (2018)	x	x	x	x	x	x	x	x	x	x
Sokmen and Kilic (2019)	x	x	x	x	x		x	x	x	x
Slišković et al. (2019)	x		x	x	x	x	x	x	x	x
Pourghaz et al. (2016)	x		x	x	x		x		x	
Hassan et al. (2019)	x	x	x	x	x	x	x	x	x	x
Suriansyah (2018)	x		x	x	x		x		x	
Carlotto and Câmara (2019)	x	x	x	x	x	x	x	x	x	x
Asaloei et al. (2020)	x		x				x	x	x	
Boström et al. (2020)	x		x	x	x	x	x	x	x	x
Glazzard and Rose (2020)	x		x	x	x	x		x	x	x
Lambert et al. (2018)	x	x	x	x	x		x	x	x	
Amri et al. (2020b)	x		x	x			x		x	
Anastasiou and Belios (2020)	x		x	x	x		x		x	
Gluschkoff et al. (2016)	x		x	x	x		x	x	x	x
van Loon et al. (2018)	x		x	x			x	x	x	
Kaynak (2020)	x	x	x	x	x	x	x	x	x	x
Platsidou and Daniilidou (2016)	x		x	x		x	x	x	x	x
Huang et al. (2019)	x	x	x	x	x	x	x	x	x	x
Tnay et al. (2020)	x		x	x	x	x	x	x	x	x
Lee et al. (2020)	x	x	x	x	x	x	x	x	x	x
Chan et al. (2021)	x	x	x	x	x	x	x	x	x	x
Poormahmood et al. (2017)	x		x	x	x	x	x		x	x
Murtedjo and Suharningsih (2016)	x	x	x	x	x		x		x	
Chakravorty and Singh (2020)	x	x	x	x		x	x	x	x	x
Wula et al. (2020)	x		x	x	x		x		x	
Alloh et al. (2019)	x	x	x	x	x	x	x	x	x	x
Janovská et al. (2016)	x	x	x	x	x	x	x	x	x	x
Wolomasi et al. (2019)	x		x	x	x		x		x	
Hascher et al. (2021)	x	x	x	x	x	x	x	x	x	x
Alvarado and Bretones (2018)	x	x	x	x	x	x	x		x	x
Fiorilli et al. (2017)	x		x	x		x	x	x	x	x
Gülbahar (2017)	x		x	x	x		x		x	
Paterson and Grantham (2016)	x	x	x	x	x		x	x	x	
Asoba and Mefi (2020)	x		x	x		x	x		x	
Gligorović et al. (2016)	x	x	x	x		x	x		x	
Wang et al. (2020)	x	x	x	x			x		x	
Berkovich (2018)	x	x	x	x	x	x	x	x	x	
Yin et al. (2017)	x	x	x	x		x	x	x	x	
de Vera García and Gambarte (2019)	x		x	x		x	x	x	x	x
Chakravorty and Singh (2022)	x	x	x	x	x		x	x	x	
Marić et al. (2020)	x		x	x	x	x	x	x	x	x
Breevaart and Bakker (2018)	x	x	x	x	x	x	x	x	x	x
García-García et al. (2021)	x	x	x	x	x		x	x	x	
Kongcharoen et al. (2020)	x	x	x	x	x		x	x	x	
Thomas et al. (2019)	x	x	x	x	x	x	x	x	x	x
Munandar et al. (2019)	x		x	x	x		x		x	
Deffaveri et al. (2020)	x		x	x		x	x	x	x	x
Mankin et al. (2018)	x	x	x	x	x	x	x	x	x	x
Ribeiro et al. (2020)	x		x	x	x	x	x	x	x	x
Ravalier and Walsh (2018)	x	x	x	x	x	x	x	x	x	x
Kuwabara et al. (2021)	x		x	x	x	x	x	x	x	x
Richards et al. (2016)	x	x	x	x	x	x	x	x	x	x
Sasmoko et al. (2017)	x	x	x	x	x				x	
Pressley (2021)	x	x	x	x	x	x	x	x	x	
Amri et al. (2020a)	x		x	x			x		x	

#### 2.1.5 Data synthesis

Overall, 61 papers were included in the final review and relevant content was summarized and categorized according to the applied teacher wellbeing approaches, namely professionalism, positivity/ flourishing and/or negativity/ deficiency. The categorisation comes from Ozturk et al. (under review) recent work on the conceptualisation of teachers’ wellbeing. The first reviewer assessed identified studies independently and applied categorisation. Although the whole process of data analysis did not apply fully a team-based approach, all disagreements or ambiguities were resolved through discussion among the first and second reviewers. The main strategy in data synthesis to resolve disagreements was consensus coding, where coders discuss differences in coding to reach a consensus (Chinh et al., 2019). Overall, we supported this process with strategies to enhance trustworthiness, such as researcher collaboration and consensus. The results are presented in Table 2.

**TABLE 2:** Categorisation of included studies.

No.	Included papers	Quotes	Applied teacher wellbeing approaches		
			Professionalism	Positivity/ Flourishing	Negativity/ Deficiency
1	Shaukat and Nazir (2017)	“… explore the **job satisfaction** of public primary school teachers and consequently find their **job commitment** …”	X		
2	Al-Asadi et al. (2018)	“… aimed to determine the level of self-reported **burnout** and the main sources of burnout among primary school teachers …”			X
3	Fareza and Tentama (2020)	“… analyze the validity and reliability of the construction of **job satisfaction**, and to find dimensions and indicators that can shape the construction of job satisfaction …”	X		
4	Abdullah and Ismail (2019)	“… re-examine and re-confirm that the **measurement model** for **job** **stress** construct with the respective dimensions and items would hold for teachers in the primary schools …”	X		X
		“The study proposed a measurement model which described factors contributing to job stress among teachers in primary schools and its respective measuring items, such as **students’ misbehavior, workloads, professional recognition, time and resource constraint, interpersonal relationship, training and support toward technology, curriculum facilities and exposure constraints and technology literacy**.”			
5	Jalali and Heidari (2016)	“… aimed to investigate the relationship between **happiness**, **subjective well-being**, **creativity** and **job performance** of primary school teachers …”	X	X	
		“Subjective well-being Scale is a 45-item questionnaire which was designed and developed by Keyes and Magyarmv to assess **emotional, psychological and social well-being**.”			
6	Smetackova et al. (2019)	“… attempts to find out what relationships between **burnout syndrome** on the one hand and **self-efficacy, coping strategies, social support,** and **job satisfaction** on the other hand exist.”	X		X
7	Yunarti et al. (2020)	“… aimed at describing the potential effect of teachers’ **work-related stress** on their **job performance** in the elementary schools …”	X		X
8	Daniilidou et al. (2020)	“… aims at elucidating their intertwining relationships by testing the hypothesis that teachers’ **resilience** mediates the effect of their **self-efficacy** on their levels of **burnout and stress**.”		X	X
		“… aimed to **test a model** describing how self-efficacy and resilience dimensions intertwine to predict stress and burnout of primary school teachers.”			
9	Edinger and Edinger (2018)	“… examine how **social capital, teacher efficacy**, and **organizational support** increase teacher **job satisfaction** …”	X		
		“… our study focuses on associations between all three of these school-based factors and their direct and indirect relationship to teacher job satisfaction. Our research **model** is presented …”			
10	Sokmen and Kilic (2019)	“… test **the model** showing **self-efficacy, autonomy, job satisfaction, engagement and burnout** levels of the teachers who work in the primary schools …”	X	X	X
11	Slišković et al. (2019)	“… focused on analyzing the relationships between several aspects of teachers’ **occupational well-being**, namely **emotions** experienced toward students, **work engagement** and **burnout**. In addition, we aimed at exploring the potential protective **role of perceived** **Principal support** as important factor in preserving these aspects of teachers’ occupational well-being.”	X	X	X
12	Pourghaz et al. (2016)	“… examine the relationship of **social support** with **organizational improvement** and **organizational effectiveness**.”	X	X	
		“… five different **dimensions of social support**, including **emotional support, instrumental support, appraisal support**, **informational support** and **social network support** were examined.”			
13	Hassan et al. (2019)	“… purpose of this research is to study the influence of selected factors such as **role conflict** factor, **role ambiguity and role overload** on **role stress** among … teachers.”	X		X
14	Suriansyah (2018)	“… aims to analyze elementary school teacher’s job satisfaction … to analyze the learning environment … and to analyze the relation between the **learning environment** and teacher’s **job satisfaction**.”	X		
15	Carlotto and Câmara (2019)	“…identify the prevalence and the predictors of the **Burnout Syndrome** …”			X
16	Asaloei et al. (2020)	“… focused on the endeavor of exploring the **work-related stress** of teachers working in the primary schools … and its eventual correlation with their **performance**.”	X		X
17	Boström et al. (2020)	“… describe how … teachers experience their **health, organizational, and social work environment, and the psychosocial safety climate in the workplace**.”	X		
18	Glazzard and Rose (2020)	“The study was based around the following three research questions: RQ1. What factors affect **teacher well-being** and **mental health?** RQ2. How does teacher well-being and mental health **impact on the progress of students?** RQ3. What **resilience strategies** are used by highly effective teachers with poor mental health to ensure that their students thrive?”	X	X	X
		“Teachers reported a number of factors that might trigger feelings of anxiety and stress, some of which were directly related to their **professional lives**, some to their **personal lives** and some that concerned **both**.”			
19	Lambert et al. (2018)	“… examined how elementary teacher appraisals of their classroom environment contribute to their risk for **stress** in the context of individual, classroom, and school characteristics, as well as state-level policy factors. … how these factors are associated with **teachers’ occupational stress, burnout**, and **commitment to teaching**.”	X		X
20	Amri et al. (2020b)	“… aimed to assess **burnout** and determine its prevalence and its risk factors …”			X
		“In this study, we tried to explain burnout through the job demands, job resources **(JD-R) model** of Schaufeli and Bakker (2004), …”			
21	Anastasiou and Belios (2020)	“…investigate the level of **job satisfaction** and **burnout** of primary school teachers …”	X		X
22	Gluschkoff et al. (2016)	“… investigate the following hypotheses: (i) high **ERI** is associated with high **burnout** and its dimensions; (ii) high ERI is associated with **poor recovery** experiences and high levels of **sleep problems**; (iii) poor recovery experiences and high levels of sleep problems are associated with high burnout and its dimensions; and (iv) the association between ERI and burnout is mediated through poor recovery experiences and sleep problem.”	X		X
		“… work stress measured in terms of **effort–reward imbalance (ERI)** has been linked specifically to **exhaustion**, the core dimension of **burnout**.”			
23	van Loon et al. (2018)	“…aims to provide insight in the prevalence and consequences of taking a more active strategy, **professional coping**.”	X		X
		“The consequent **well-being** in the form of **burnout** or its opposite **work engagement** are the result of whether these efforts have been successful. Moreover, **intentions to leave** are the final outcome …”			
24	Kaynak (2020)	“… investigation of elementary teachers’ perspectives on **well-being** and the influence of **contextual factors** on their degree of well-being through examining their experiences and stories.”	X	X	
		“The purpose of the study is to develop an in-depth understanding of factors influencing teachers’ overall **professional well-being** …”			
		“By compiling the emergent factors in these studies, it is possible to see five components of teacher well-being as shown in Figure 1. [aspects of professional well-being of teachers: sense of **self-efficacy, job satisfaction**, sense of **autonomy, reasonable workload**, and **supportive school culture**]”			
25	Platsidou and Daniilidou (2016)	“… compare measures of teachers’ **burnout** obtained by three of the most widely used instruments, namely, the ones developed by Maslach and Jackson (1986); Pines and Aronson (1988), and Kristensen et al. (2005), respectively.”			X
26	Huang et al. (2019)	“Following the recent development of the **JD-R model**, this study aims to examine how the effects of the **job characteristics** of teaching on **teacher well-being** are mediated by teachers’ **individual characteristics**, namely **self-monitoring** and **self-efficacy**.”	X	X	
27	Lee et al. (2020)	“… aimed to provide a clearer understanding of perceived **school leaders**’ **learning support** and its predictive relationships with teachers’ **psychological needs satisfaction** and **work-related outcomes**.”	X	X	
28	Chan et al. (2021)	“The present study … (a) assess teachers’ **professional well-being**, (b) examine associations between specific **job characteristics** and teacher wellbeing, and (c) explore effective practices to help bolster teachers’ professional functioning during the pandemic.”	X		X
		“To better understand how teachers **function psychologically** during the pandemic, the present study evaluates teachers’ **job satisfaction** and **emotional exhaustion** based on their teaching experience from March to June 2020 during the early stages of COVID-19.”			
29	Poormahmood et al. (2017)	“… determining relationships between **psychological well-being**, **happiness** and perceived **occupational stress** among primary school teachers …”	X	X	X
		“The scales of psychological well-being … includes six domains that measure **autonomy …, environmental mastery …, personal growth …, positive relations with others …, purpose in life** … and **self-acceptance** …”			
30	Murtedjo and Suharningsih (2016)	“… analyze the relationship between the variables of **organizational culture, work motivation** and **job satisfaction** with the **performance of teachers**.”	X		
31	Chakravorty and Singh (2020)	“… examine the mediating effects of **work–family conflict** (WFC) between **job demands** and **burnout**.”	X		X
		“Using the **conservation of resources (COR) theory**, …”			
		“The COR theory … recognizes the importance of both work and family in understanding burnout.”			
32	Wula et al. (2020)	“… describing the **performance** of elementary school teachers … and how it is predicted by their **job satisfaction**.”	X		
33	Alloh et al. (2019)	“… examine primary school teachers’ **burnout levels** … and to gain insight into teachers’ **workplace conditions** and **perceptions of their jobs**, which may provide sources for future initiatives to improve **teachers’ performance** that would directly affect students’ achievements and school competency.”	X		X
34	Janovská et al. (2016)	“… explore the relationship between the **supportive behavior of the head teacher** and selected **personality traits** in relation to the emotional component of **subjective well-being** (positive and negative emotions) and its cognitive component (overall life satisfaction, satisfaction with work) of primary school teachers…”	X	X	X
		“Conceptually, this research project was based on the **hedonic** perspective to **subjective well-being**, which was operationalized by life satisfaction and frequency of experiencing positive and negative emotions.”			
		“Within the context of **teachers’ well-being** this study was mainly focused on **work satisfaction** but the related concepts of **overall life satisfaction** and **emotional well-being** were also analyzed here.”			
35	Wolomasi et al. (2019)	“… describe the **job satisfaction** of elementary school teachers … and how it predicts their **job performance**.”	X		
36	Hascher et al. (2021)	“… aims at understanding how changes in teachers’ professional lives that were related to **school closure** affected Swiss primary **teachers**’ **professional well-being**.”	X		
		“The present study focuses on the results of the following questions: 1. How would you rate your well-being at work out of 10 (1 = extremely **low**; 10 = extremely **high**) at the moment? What has led you to make that judgment? How typical is that rating of how you usually feel at work? 2. Has there been a time during the COVID-19 crisis when you would have rated your sense of well-being at work at a low level? Can you tell me what was happening at that time? …”			
37	Alvarado and Bretones (2018)	“… to identify and characterize the **working conditions** and **new stressors** in teachers 35 years after the theory formulated by Blasé (1982).”	X		
		“… to understand the teachers’ perceptions of their **profession**, associated **risks**, and **wellbeing** implications.”			
38	Fiorilli et al. (2017)	“… to analyze whether the **multidimensional construct of teacher burnout** mediated the relationship between **emotional competence** and **social support**. … aimed to test the relationships among all of these variables in a single model.”	X	X	X
39	Gülbahar (2017)	“… to reveal the relationship between elementary school teachers’ perceptions of **work engagement** and perceptions of **organizational trust**.”	X		
40	Paterson and Grantham (2016)	“… to explore the shared **understanding of teacher wellbeing** (TWB) and the **factors** that may support it positively. An **ecological framework** allows …”	X	X	
		“… completing the Glasgow Motivational and Wellbeing Profile. This provided a profile of wellbeing for each school—highlighting levels of **affiliation, agency** and **autonomy**, as well as how **healthy** and **safe** participants felt within their team.”			
41	Asoba and Mefi (2020)	“… to determine the **stress** challenges faced by teachers in schools.”			X
42	Gligorović et al. (2016)	“… to explore whether **school culture** observed through three aspects: Teacher Professionalism and Goal Setting, Professional Treatment by the Administration and Teacher Collaboration has an impact on the dimensions of **job satisfaction** of primary teachers …”	X		
		“… to understand the relations between school culture aspects and teacher job satisfaction …”			
43	Wang et al. (2020)	“… aims to compare teacher stress in China and the United States under the **perspective of the Classroom Appraisal of Resources and Demands (CARD)**. … comparing **teacher stress** across eastern and western countries (such as China and the United States), the findings will extend our understanding of teacher stress internationally … through the lens of appraisal of demands and resources in classrooms …”			X
44	Berkovich (2018)	“… study of **teachers’ trust in their principal** from a multidimensional perspective.”	X	X	
		“… focuses on the classification of various types or profiles of **trust relationships** in schools.”			
		“…also investigated how these types of trust relations correlate with **teachers’ wellbeing** and their inclination to make an extra effort.”			
		“…, teachers’ wellbeing indicates their **socio-emotional** state. In the present work, I chose to focus on **teachers’ relational wellbeing** (i.e., the affect in interactions with the principal), …”			
45	Yin et al. (2017)	“…this study adopts Grandey’s (2000) **integrative model of emotional labor** …”	X	X	
		“… examines the relationships between the **emotional demands** of teaching, **teachers’ emotional labor strategies**, and **teacher efficacy**.”			
46	de Vera García and Gambarte (2019)	“… to establish relationships between the dimensions of **burnout** and the **resilience perceived** …”		X	X
		“Burnout: syndrome of physical and emotional exhaustion, which implies the development of negative attitudes toward work, poor self concept and loss of interest in the activities carried out. **Three subvariables**:			
		a. Exhaustion or emotional fatigue: …			
		b. Cynicism or depersonalization: …			
		c. Efficacy or personal fulfillment:			
		Resilience or positive adaptation to circumstances of significant adversity: an essential component for good work performance and a basic element for the protection of workers’ welfare. **Five subvariables** or dimensions: personal competence, self-demand and tenacity; confidence in one’s intuition and tolerance of adversity; positive acceptance of change and secure relationships; control; spiritual influences.”			
47	Marić et al. (2020)	“…to estimate the prevalence of **burnout** syndrome … identify the factors associated with burnout syndrome, …”			X
48	Breevaart and Bakker (2018)	“Using **job demands–resources (JD-R) theory**, … integrates **the challenge stressor-hindrance stressor framework** and **leadership theory** to investigate the relationship between **daily transformational leadership behavior** and employee **work engagement**.”	X		
49	García-García et al. (2021)	“…to analyze the level of **competence** perceived … as a function of contextual variables.”	X		
50	Kongcharoen et al. (2020)	“… to explore the levels of **stress** and **work motivation** … and to investigate factors affecting stress of teachers …”	X		X
51	Thomas et al. (2019)	“Drawing theoretically from **the social network perspective** …”	X		
		“…to explore beginning teachers’ (BTs’) **network** and how this is related to their **job attitudes**, as important precursors of **teacher retention**.”			
52	Munandar et al. (2019)	“The **teacher’s professional competence** is influenced by several factors, including **welfare** and **motivation** to teach. … to analyze the influence of these factors.”	X		
		“…the involvement of the **Principal** is important in this research.”			
53	Deffaveri et al. (2020)	“…to investigate the presence of symptoms of **anxiety** and **stress** …”			X
54	Mankin et al. (2018)	“… focuses on two core **positive** aspects of **teacher wellbeing**: **teaching efficacy** and **school connectedness**.”	X		
55	Ribeiro et al. (2020)	“…to investigate the association between burnout syndrome (BS) and the **occupational characteristics** of primary and secondary school teachers …”	X		X
56	Ravalier and Walsh (2018)	“… to investigate **stress** in this sample, and to establish which **work-related factors** most influenced the experience of stress …”	X		X
57	Kuwabara et al. (2021)	“…to explore the association between **overwork** and **mental stress** …”	X		X
58	Richards et al. (2016)	“Grounded in **role theory** … to examine the impact of **resilience** on perceived **stress** and **burnout** …” “…to evaluate a research-based **conceptual model** for understanding the relationships among resilience, role stress, and burnout …”		X	X
59	Sasmoko et al. (2017)	“…to examine the **subjective well-being** condition of teachers … and identify the most **decisive indicator** forming of the teacher’s subjective well-being.”		X	
		“The instrument had been made based on two **subjective** wellbeing theories by Diener.”			
60	Pressley (2021)	“With all the new challenges and **COVID-19** policies teachers faced …… to explore how the new teaching approaches and requirements have impacted elementary teachers’ **efficacy**, specifically **instructional and engagement efficacy**.”	X		
61	Amri et al. (2020a)	“… to determine the prevalence and risk factors of **burnout** …”			X

## 3 Results

This current study has three aims; (1) to assess the extent to which existing research reflects the multidimensional nature of the term teacher wellbeing, (2) to determine whether a holistic construct of teacher wellbeing could be justified, and (3) to evaluate the methodological quality of studies identified. Consequently, the following section outlines the results of the study selection process, quality assessment, and the categorization of teacher wellbeing.

### 3.1 Study selection and characteristics

**FIGURE 3:**
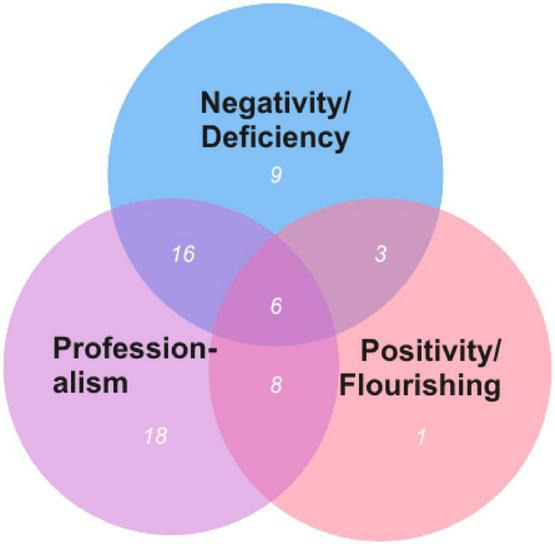
Teacher wellbeing approaches and total numbers for each category.

The review process identified 61 eligible studies from the 1,676 studies gathered through the initial searches. In all of the 61 studies examined, school levels comprised solely primary/elementary education samples or subsamples (e.g., in the case of comparison studies). If the results were presented separately for the subsamples (e.g., primary-level teachers from a sample of mainstream school teachers), those papers were included, otherwise, excluded. All types of research and design were included apart from meta-aggregation by which secondary summaries of data are provided (e.g., meta-analysis/ systematic reviews) and intervention studies. The reason for that these studies do not examine/ conceptualize teacher wellbeing directly. All included studies were synthesized and then categorized according to teacher wellbeing approaches (see Figure 3).

### 3.2 Quality analysis results

The full quality of reporting and replicability assessment against relevant quality assessment criteria is presented in Table 1.

Among all the reviewed studies (61 in total), 24 met all criteria or all of the essential criteria which means 39.3% of the studies have met all necessary and desirable quality requirements in terms of methodological quality. However, the results highlight areas which are poorly reported in terms of essential criteria, including the data collection process, sampling, and ethics. Ethics and data collection are the most problematic ones in terms of essential criteria, respectively, 45.9% (28) and 40.9% (25) of the included studies have not met these criteria. A detailed examination of the quality of studies underpinning research for each of the (sub)categories can be found in Supplementary Appendix 2.

### 3.3 Individual study categories and results

Individual study categories according to the applied teacher wellbeing approaches which are negativity/ deficiency, positivity/ flourishing, and/or professionalism are presented in Table 2.

All of the 61 studies that were reviewed met at least one of the teacher wellbeing approaches, but some met two (16+3+8) or even three (6). Professionalism was the most prevalent approach. In total, 48 of the listed papers were evaluated in part or entirely under this category. 18 of the 48 papers were categorized entirely under the professionalism approach. This was followed by the negativity/ deficiency approach. In total, 34 of the examined studies applied partly or fully this approach. Of them, while 9 of them were solely categorized under the negativity/ deficiency approach, 16 take into consideration of both professionalism and the negativity/ deficiency approach. The least prevalent category was the positivity/ flourishing approach with 18 of the included studies included in this category. It was found that only one study solely focused on the positivity/ flourishing approach.

In the examination of the multidimensionality of the studies under the main and sub-categories, 28 (18+9+1) of the reviewed studies took into consideration only one approach when conceptualizing teacher wellbeing. In other words, almost half of the listed studies (45.9%) conceptualized teacher wellbeing as single-dimensionally. The remaining 27(16+8+3) (44.2%) studies take into account binary combinations of them, and 6 studies refer to all of them while examining teacher wellbeing. The most important thing is there are only 6 studies that have all three of them together which means only 9.8% of the reviewed studies conceptualize teacher wellbeing multi-dimensionally. Table 3 demonstrates all the categories and subcategories with numbers and percentages.

**TABLE 3:** Teacher wellbeing approaches and total numbers for each main and subcategory.

Teacher wellbeing approaches	Professionalism	Positivity/ flourishing	Negativity/ deficiency
Professionalism	18	8	16
Positivity/ flourishing		1	3
Negativity/ deficiency			9

The intersection of all three approaches (Professionalism approach + Positivity/ flourishing approach + Negativity/ deficiency approach) contains 6 papers.

• Only Professionalism approach (18 = 29.5%).
• Professionalism approach + Negativity/ deficiency approach together (16 = 26.2%).
• Only Negativity/ deficiency approach (9 = 14.7%).
• Professionalism approach + Positivity/ flourishing approach together (8 = 13.11%).
• All together–Professionalism approach + Positivity/ flourishing approach + Negativity/ deficiency approach together (6 = 9.8%).
• Positivity/ flourishing approach + Negativity/ deficiency approach together (3 = 4.9%).
• Only Positivity/ flourishing approach (1 = 1.6%).

## 4 Discussion

The aim of the current study was three-fold; (1) to assess the extent to which existing research reflects the multidimensional nature of the term teacher wellbeing, (2) to determine whether a holistic construct of teacher wellbeing could be justified, and (3) to evaluate the methodological quality of studies identified. The review found that to date, research has predominately (but not exclusively) adopted a professionalism approach as the dominant discourse in defining teacher wellbeing. However, a crucial finding of the review is that only a small portion of the identified literature recognizes teacher wellbeing as a multidimensional construct, with only six papers identified as including the three main discourses in one analysis. This finding raises questions about the extent to which research into teacher wellbeing takes account of a holistic perspective (referring to three main discourses together). We believe that by considering various dimensions such as professionalism, positivity/flourishing, and negativity/deficiency together, a more holistic understanding of teacher wellbeing can be attained. The proposed broader perspective enables us to recognize the interplay between personal and professional elements, as well as the reciprocal relationship between the state of the teaching profession and the wellbeing of teachers (Ozturk, 2023).

Another significant contribution of this paper is evaluating the quality of the published papers. Assessing the quality of published papers is crucial for determining the extent to which confidence can be placed in findings contributing to our understanding of teacher wellbeing. The findings of the quality assessment showed that there is room for improvement in the reviewed studies in terms of ethical standards and data collection processes. The rest of this section will seek to discuss these findings in detail.

### 4.1 Conceptualisation of primary school teachers’ wellbeing

Although there is no single agreement establishing what should be included in a definition of teacher wellbeing, there is agreement that it is a multidimensional, complex construct. The current review revealed that there is an emphasis (or bias) within research, investigating the phenomena through the lens of a single approach, namely professionalism which focuses entirely on the work domain. This approach mainly conceptualizes teacher wellbeing through self-efficacy, job satisfaction, work engagement, authority, and competence. This finding is not consistent with the results of the earlier reviews and common understanding within the literature. In their review in Spilt et al. (2011) found that the term wellbeing has largely been examined through a focus on stress and burnout which means the negativity/ deficiency approach. Similarly, Roffey (2012) states that teacher wellbeing is typically examined through the lens of negativity. Given earlier reviews predate the current study by a decade, this indicates a likely change in trends regarding conceptual understanding in how teacher wellbeing is understood but critically does little to move toward greater holism in its definition, choosing to supplant, rather than embrace alternative discourses.

According to the findings of the current review, the second most common approach is a negativity/ deficiency approach which focuses on negative feelings of stress, burnout, anxiety, depression, and other associated constructs. Respectively 26% of the studies applied professionalism and negativity together and almost 15% applied solely the negativity/ deficiency approach. Although this combination (professionalism and negativity/ deficiency together) somehow reflects the idea of multidimensionality, this also reflects the sense of consistency of applying the professionalism approach while conceptualizing teacher wellbeing. Therefore, this finding also reinforces the singular dominant perspective. In regard to this, more recently Viac and Fraser (2020) have paid attention to working conditions which shape teachers’ wellbeing. However, we believe, while heavily focusing on one aspect i.e., professionalism, important information is lost by neglecting alternative lenses.

Regarding the findings on professionalism and negativity/ deficiency approaches, it is also important to recognize that teachers are not immune to stressors outside of work. These stressors have the potential to harm their general wellbeing, which in turn can have repercussions for their work performance and their wellbeing in schools. Furthermore, as stated earlier by Warr (1999) work-related wellbeing and general wellbeing influence each other. Therefore, it is essential to comprehend not only the professional factors influencing teacher wellbeing but also the external determinants that can impact it.

Although relatively limited, almost half of the papers conceptualize teacher wellbeing using two approaches together. When examining combinations of the approaches, the combination of professionalism and negativity/ deficiency approach (26%) appears as a prevalent combination, followed by the combination of professionalism and positivity/ flourishing (13%). These results strengthen the idea of domination of the professionalism approach and also indicate how the positivity/ flourishing approach is insufficiently covered within the literature. Results revealed that only one paper conceptualizes teacher wellbeing solely focusing on the positivity/ flourishing approach. In short, although the combinations intensify the idea of a need for further multidimensionality as identified combinations present a two-dimensional spectrum that arguably does not accurately reflect the nature of the term.

A further significant finding of this review is only 6 papers were identified that conceptualize teachers’ wellbeing in the “fullest” holistic sense, utilizing all three major discourses. We examined 61 papers in total and found that only almost 10% of these conceptualize the term mirroring its multidimensional, complex construct. Although the literature agrees that teacher wellbeing is a multidimensional construct, this does not appear to be reflected in current research. The multidimensionality of the wellbeing was confirmed by many scholars, for instance, Ryff’s (1989) wellbeing model, Seligman’s (2011) PERMA framework etc., however, the current literature of teacher wellbeing does not reflect this situation. Recently, Hascher and Waber (2021) highlighted the complex and multidimensional nature of teacher wellbeing, emphasizing the need to integrate both positive aspects (e.g., positive affect, satisfaction) and negative aspects (e.g., negative affect, worries, stress) in its conceptualisation. Aligned with this perspective, the current study advocates for an accurate investigation of teacher wellbeing, necessitating a multifaceted approach considering various dimensions such as contextual elements arising from the profession or school environment, as well as both positive and negative aspects.

In their previous review, Hascher and Waber (2021) taking a multidimensional approach to defining teacher wellbeing broadens the options to define or conceptualize the term. Yet, we believe this intention should be interpreted carefully since they initially decided to restrict their search keywords to wellbeing solely. Because of the restriction on their search terms, they might not have ended up with a comprehensive understanding and ultimately have missed the multidimensional approach to teacher wellbeing. As opposed to this restricted application, we applied more comprehensive search terms to have a holistic understanding of the term.

Here we would like to slightly touch on one point: the jingle-jangle fallacies. We are aware that both studies’ findings might be affected by the jingle-jungle fallacies within the literature. As stated before, there is no consensus on what influences teacher wellbeing and what forms a component of teacher wellbeing. Together with this unclarity, the jingle-jangle fallacies make it harder to make comments on the findings of both our study and the previous reviews. We are not going to discuss these in detail here (since it is not the priory aim of this paper), but we should state that we are aware of these limitations. Nevertheless, Hascher and Waber (2021) argue that the definition and the operationalization of teacher wellbeing differ. Although here we did not explicitly investigate the definition of teacher wellbeing, we can confirm that the conceptualisation and/or operationalisation of the term differs. And moreover, our findings confirm that the conceptualisation of the term does not actually reflect the multidimensionality of the construct.

For instance, if a study conceptualizes teacher wellbeing as professionalism and looks into job commitment and job satisfaction (see Shaukat and Nazir, 2017), it simply means that the study’s findings are only applicable to the subdimensions that were specifically looked at, not to teacher wellbeing as a multidimensional construct. On the contrary, for example, Slišković et al. (2019) investigated teachers’ occupational wellbeing and looked at emotions experienced toward students, work engagement, and burnout. All of these subdomains point out another approach in terms of conceptualisation of teacher wellbeing such as work engagement refers to professionalism, but burnout refers to negativity. This means this study considers all three approaches while conceptualizing teacher wellbeing and reflects the multidimensionality of the term.

On the other hand, although in their study Slišković et al. (2019) stated that they are investigating teachers’ occupational wellbeing which implies close connotation with professionalism, they conceptualize the term much more holistically in fact. However, based on the findings of the current research, there are surprisingly few studies of this kind that examine teacher wellbeing in the context of a holistic approach.

As a result, we could say our findings are somehow consistent with the previous review of Hascher and Waber (2021) in terms of significant heterogeneity in teacher wellbeing approaches, but these approaches are not fully multidimensional in fact. Acknowledging the multifaceted aspects of teacher wellbeing–including positive, negative, and professional dimensions–is essential in investigating teacher wellbeing. When there is a lack of agreement among concepts used to explore teacher wellbeing, it becomes challenging to identify which specific factors are most strongly associated with each construct and which interventions are most effective in mitigating their effects. Such clarity is crucial as it would facilitate more targeted and effective strategies for supporting teacher wellbeing in educational settings.

This research holds significant implications for policymakers, especially given that policy documentation frequently focuses on stress and burnout (negativity discourse). Shedding light on the multifaceted nature of teacher wellbeing, could offer valuable insights into how policymakers assess and address the needs of teachers. Ultimately, such insights could pave the way for more comprehensive and nuanced approaches to supporting teacher wellbeing at a policy level. Therefore, it is crucial to interpret research findings in this field with careful consideration of their broader implications.

### 4.2 Differences in the quality of studies underpinning research

The process of determining what is known through research in regard to various research questions, also known as research synthesis, entails making judgments of the quality and relevance of the research findings taken into account (Gough, 2007). In this review, each included article’s methodological quality was evaluated with a checklist adapted from Croucher et al. (2003). Among a total of 61 papers, 24 studies had met all criteria or all of the essential criteria. In other words, more than one-third of the included studies (39.3%) had methodologically met all essential and desirable quality requirements, indicating that a good amount of evidence was of satisfactory quality. At the same time, almost every (sub)category’s (almost) half of it is methodologically excellent.

The findings, however, draw attention to areas that, in terms of essential criteria, are underreported/underdeveloped. These include the data-gathering procedure, sampling, and ethics. From these, ethics is the most problematic essential criterion. The reason for this may be it was not considered essential criteria for some studies like examining measurement models. Nonetheless, it should be clearly reported if the authors take into consideration any ethical matters. Similarly, data collection is the second highlighted one as not meet the quality criteria. This situation raises some questions about the robustness of the studies.

Identifying the quality of the published papers is essential to identify how the findings can contribute to our understanding. For instance, when the information of low quality of a paper is missing, readers might be misinformed about the impact and relevance of the paper. Based on the contribution of our paper, we argue that findings highlight a weakness inherent to the existing research base. We urge authors of future studies to be more detailed in their work to strengthen the robustness of the research base.

## 5 Limitations and future directions

Although our study complied with the PRISMA framework for systematic literature reviews, there are still some limitations. The first limitation is about the scope of the review. Even though we made an effort to be comprehensive, we might have missed certain related concepts like enthusiasm. Nevertheless, it should be clearly stated that we included more than 20 terms, such as burnout, job satisfaction, resilience, etc., which might be especially relevant.

A second limitation is more particularly related to the risk of bias and includes the database choice, omitting gray literature, and the use of the sole publication language. Furthermore, we might have overlooked certain findings that could have added to our understanding of teacher wellbeing because we did not include gray literature in our review. Therefore, we admit that our inclusion and exclusion criteria may have resulted in the exclusion of pertinent literature (as in any systematic review). Yet, we believe all of these limitations are somehow inescapable, therefore, the extent of these limitations is relatively minor.

Our review identifies several critical areas for further studies. First, we recommend doing additional analysis for studies explicitly on wellbeing to have a deeper understanding of the conceptualisation of teacher wellbeing. We examined all the included studies together, however, some studies declare that they are solely and directly focused on teachers’ wellbeing. Therefore, we believe looking at those studies could give us much more focused understanding in terms of the conceptualisation of teacher wellbeing. Furthermore, checking teacher wellbeing’s interrelatedness to other constructs such as mental health, resilience, etc. could promote an in-depth understanding of the term.

## 6 Conclusion

This systematic review indicates that teacher wellbeing is an important and developing study subject. Findings illustrate that teacher wellbeing is dominantly conceptualized with the professionalism approach. Results consistently reflect the dominant application of the professionalism approach while conceptualizing teacher wellbeing. However, this is not completely consistent with the concerning body of literature that focuses on stress and burnout (negativity/ deficiency approach) while exploring teachers’ mental health and wellbeing. Moreover, this finding points clearly to the missing domains in the research base (i.e., positivity/ flourishing approach).

The most significant finding is that very few articles include all three domains (only 6 papers identified). The fundamental critique of the previous reviews and the field, in general, is that the field is failing to take a comprehensive approach to teacher wellbeing despite a general agreement with respect to significant heterogeneity in teacher wellbeing approaches. This study argues that important information is lost through neglecting alternative lenses, requiring further attention in order to address teacher wellbeing fundamentally. Therefore, research should be holistic which means future papers should explicit multiple major discourses when examining teacher wellbeing.

Moreover, we believe simply including all three domains in research is not enough; perspectives on wellbeing must be thoroughly examined and integrated into future studies in order to provide a comprehensive understanding of teacher wellbeing. This entails not only acknowledging the existence of these domains but also delving deeply into their implications and interrelationships within the context of teacher wellbeing. In doing so, researchers can avoid overlooking important aspects of teacher wellbeing and contribute to a more holistic understanding of the subject.

The multidimensionality of the construct and the diversity of approaches, on the other hand, necessitate a solid knowledge base on which future research and practice can build. In terms of our knowledge of teacher wellbeing conceptualisation, agreeing on its core elements, such as a predominance of professionalism aspects, as well as deeper linkages to the qualities and problems of the teaching profession, may assist in overcoming contradictions. This, in turn, informs future studies and practices to promote teacher wellbeing.

## Data availability statement

The original contributions presented in this study are included in the article/Supplementary material, further inquiries can be directed to the corresponding author.

## Author contributions

MO: Conceptualization, Data curation, Formal analysis, Funding acquisition, Investigation, Methodology, Project administration, Resources, Validation, Visualization, Writing – original draft, Writing – review & editing. MW: Supervision, Writing – review & editing. GS: Supervision, Writing – review & editing.
