# Supplementary material for: A systematic review of primary school teachers’ wellbeing: room for a holistic approach
Source: Front Psychol. 2024 Jun 10;15:1358424. doi: 10.3389/fpsyg.2024.1358424 (PMC11195814; doi:10.3389/fpsyg.2024.1358424)
Supplement: Supplementary file 1 [file Data_Sheet_1.docx]

Appendix 1

SPIDER Framework

|  | INC | EXC |
| --- | --- | --- |
| Sample  Which type of participants are of interest? | In-service primary/elementary school teachers who are responsible for ages 5 to 12 years of age in mainstream or publicly funded schools  For studies that include this eligible cohort as part of a wider sample, retain for the next stage of filtering. | Teachers in specialist or private setting  Out of primary level e.g. post-primary, pre-primary, secondary  Out of mainstream e.g. private schools  External coaching/ employment (e.g. external subject specialist – across multiple settings) |
| Phenomenon of Interest  Which behaviours, decisions, experience are being considered? | Wellbeing  The focus must be on teachers' perceptions of phenomena of interest (i.e. teachers reporting of wellbeing).  This includes positive perceptions as well as deficit approaches.  This may include, but is not limited to the following terms:  workload, burnout, stress, recognition, job satisfaction, self-efficacy, autonomy, competence, support, engagement, mental health, happiness, resilience | Factors that predict of influence wellbeing e.g. financial insecurity should be rejected.  Teachers’ discussions of others wellbeing e.g. pupils should be rejected. (NB: Teachers’ discussing other teachers’ wellbeing would be included)  Reports of school climate, ethos, or culture should be rejected. |
| Design  Theoretical framework or research method | Case study, cross-sectional, survey, longitudinal, empirical etc.  Auto-ethnography, Psychometric validation  EFA and/or CFA analysis in which items from the same or different measures are combined to explore their associations  Pilot study | Meta-aggregation by which secondary summaries of data are provided (e.g. meta-analysis/ systematic reviews)  Correlation, regression studies that do not examine/ conceptualise teacher wellbeing directly  Intervention e.g. programs, trainings even directly related to TWB |
| Evaluation  Are there specific outcomes to be considered? Even more subjective ones such as views, attitudes, etc. | Teachers’ views/ experience on their wellbeing, subjective wellbeing, work place wellbeing etc.  Studies that example wellbeing through a psychological needs model  Studies that report on or adopt models of teacher wellbeing directly | Teachers’ commentary on others’ wellbeing e.g. students |
| Research type  Qualitative, quantitative or mixed methods | All types; qual, quant or mixed | Meta-aggregation by which secondary summaries of data are provided (e.g. meta-analysis/ systematic reviews) |

Appendix 2

**Comparison of Study Quality Assessment**

***Only Professionalism approach (18)***

In this group, 7 of the studies met all criteria or all of the essential criteria which means less than half of the studies have excellent quality in terms of methodological quality. On the other hand, half of the studies (9) and more than half of them (11) could not meet some essential criteria, respectively data collection and ethics criteria. These two essential criteria were the most problematic ones in terms of methodological quality criteria for this group. Together with these, although reflexivity and theoretical perspective are not the essential criteria, they were the most problematic ones among the desirable criteria cause respectively 11 and 7 papers were not able to meet these criteria.

***Professionalism approach + Negativity/ deficiency approach together (16)***

In this group, 7 of the studies met all criteria or all of the essential criteria which means almost half of the studies have really good quality in terms of methodological quality. However, like in the previous group, half of the studies (8) and slightly less than half of them (7) could not meet some essential criteria, respectively data collection and ethics criteria. These two essential criteria were the most problematic ones in terms of methodological quality criteria for this group, too. In terms of desirable criteria, again, the theoretical perspective criterion comes forward. 7 papers could not be able to meet this desirable criterion in this group.

***Only Negativity/ deficiency approach (9)***

In this group, 3 of the studies met all criteria or all of the essential criteria which means the one of third of the studies have very good quality in terms of methodology. Yet, more than half of the studies (6) and half of them (4) could not meet some essential criteria, respectively sampling and ethics criteria. These two essential criteria are the most controversial ones in terms of methodological quality criteria for this group. On the other hand, as desirable categories, theoretical perspective and reflexivity come one step forward since 7 and 4 of the studies from 9 listed studies could not meet these ones respectively.

***Professionalism approach + Positivity/ flourishing approach together (8)***

Less than half of the listed studies (3) under this group met all criteria or all of the essential criteria. Also, data collection and ethics take attention as the most controversial essential criteria. Respectively, 3 and 5 of the papers failed to meet these criteria which means that more than half of the studies were problematic in terms of the ethics criterion.

***All together - Professionalism approach + Positivity/ flourishing approach + Negativity/***

***deficiency approach together (6)***

Of the 6 studies in this group, 3 studies met all criteria or all of the essential criteria means that exactly half of the studies under this category have excellent methodological quality. The theoretical perspective desirable criterion was the noticeable criterion for this category, and four research were unable to achieve this. On the one hand, with one study for each criterion, sampling, data collection, and data analysis appear to be not fully achieved in terms of essential criteria.

***Positivity/ flourishing approach + Negativity/ deficiency approach together (3)***

Only one study under this group met all criteria. 2 of the studies could not meet sampling (E) and theoretical perspective (D) criteria.

***Only Positivity/ flourishing approach (1)***

There is only one paper under this category. This paper failed to meet some essential criteria namely, sampling, data analysis, and ethics.
